# Insect symbionts in food webs

**DOI:** 10.1098/rstb.2015.0325

**Published:** 2016-09-05

**Authors:** Ailsa H. C. McLean, Benjamin J. Parker, Jan Hrček, Lee M. Henry, H. Charles J. Godfray

**Affiliations:** 1 Department of Zoology, University of Oxford, South Parks Road, Oxford OX1 3PS, UK; 2 Faculty of Earth and Life Sciences, University of Amsterdam, De Boelelaan 1085–1087, 1081 HV Amsterdam, The Netherlands

**Keywords:** food web, symbiont, symbiosis, aphid, mutualism, resistance

## Abstract

Recent research has shown that the bacterial endosymbionts of insects are abundant and diverse, and that they have numerous different effects on their hosts' biology. Here we explore how insect endosymbionts might affect the structure and dynamics of insect communities. Using the obligate and facultative symbionts of aphids as an example, we find that there are multiple ways that symbiont presence might affect food web structure. Many symbionts are now known to help their hosts escape or resist natural enemy attack, and others can allow their hosts to withstand abiotic stress or affect host plant use. In addition to the direct effect of symbionts on aphid phenotypes there may be indirect effects mediated through trophic and non-trophic community interactions. We believe that by using data from barcoding studies to identify bacterial symbionts, this extra, microbial dimension to insect food webs can be better elucidated.

This article is part of the themed issue ‘From DNA barcodes to biomes’.

## 1. Introduction

Symbiotic associations with microorganisms are now recognized to be widespread among insects and to have many important effects on their biology [1,2]. Despite this, community ecologists have paid relatively little attention to the role symbionts might have in the structure and dynamics of insect-based food webs. Conversely, symbiont biologists seeking to understand how carrying a microorganism might affect a host's interactions with competitors and natural enemies have predominantly focused on interactions between pairs of species rather than considering the net effects of multiple interactions in a wider food web context. Of course, in an emerging field, where new associations and new phenomena are being continually discovered, it makes perfect sense to begin with two-species interactions and not to complicate food web studies before there is a strong argument it is necessary. We argue here that this time has come, and that considering the community ecological implications of this type of interaction is the next logical step in understanding the biological importance of symbiotic microorganisms. In this review, we will illustrate the potential for, and necessity of, including symbionts in future food web studies, and make a case for using barcoding studies for this purpose. We focus in particular on aphids, a group whose community ecology and symbiont biology are relatively well studied [3–5]. We first briefly introduce this system, before discussing how the known functional effects of symbionts might influence community interactions and the structure of food webs.

### (a) Aphids as model systems for studying food webs and symbiosis

**Figure 1.**
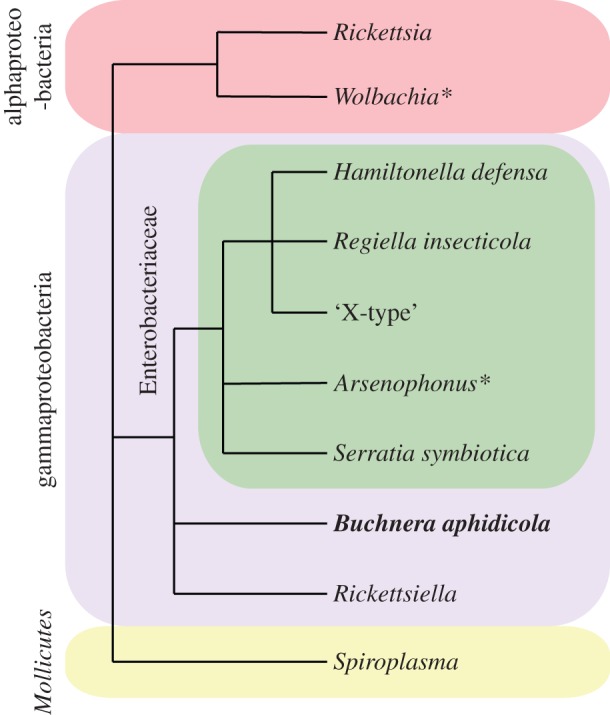
Taxonomic relationships of aphid bacterial symbionts. The asterisks refer to species of symbionts not found in pea aphids. The primary symbiont, present in virtually all aphids, is in bold type.

The structure and dynamics of source food webs based on aphids (Aphidoidea) have been extensively studied, and this group has also emerged as a model system to explore the biology of obligate and particularly facultative symbionts. Almost all aphids possess an obligate (or primary) nutritional symbiont, *Buchnera aphidicola*, which synthesizes amino acids and other essential nutrients absent in its phloem diet [6]. In addition, aphids host a number of facultative (or secondary) symbionts that are not essential for host survival and typically are found in only a fraction of the individuals in a population [7–13] (figure 1). The species that has received the most attention is the pea aphid (*Acyrthosiphon pisum*), which harbours at least seven species of secondary bacterial symbiont [5] (figure 1). Secondary symbionts that are predominantly maternally inherited can spread by one of two broad strategies: manipulation of their host's reproduction so that the symbiont is transmitted to more offspring than is possible via simple transmission to daughters, and through the provision of absolute or conditional fitness benefits for their hosts. Both reproductive manipulation [14] and fitness enhancement have been reported for aphids, with the latter being by far the most important. Below we shall review these symbiont effects on host biology though we note here that while there have been a few studies of the patterns and prevalence of symbiont infections in the field [15–17], most studies (including experimental investigations of the effect of symbionts on aphid phenotype) have been carried out in the laboratory. Accordingly, these studies have not considered how symbionts might be influenced by a more stressful environment in the field and by the presence of multiple possible natural enemies.

Aphids are an excellent model system in food web ecology because they are exploited by several species-rich guilds of natural enemies, and because they are relatively easy to manipulate in the field [18]. They are attacked by generalist predators (including many insects and insectivorous birds) though the majority of predation is by specialists such as ladybirds (Coccinellidae), hover flies (Syrphidae) and predatory midges (Cecidomyiidae). Two major clades of parasitoids have evolved to attack aphids (Aphidiinae in the Braconidae and *Aphelinus* in Aphelinidae) and the parasitoids themselves are attacked by a variety of specialized hyperparasitoid groups [19]. Finally, aphids are infected by a number of fungal pathogens, some of which are aphid specialists [3,4].

**Figure 2.**
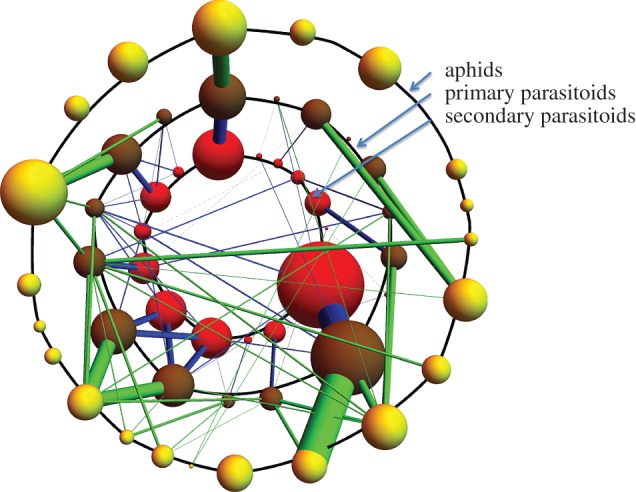
Quantitative food web describing the interactions between aphids and their parasitoids and hyperparasitoids. The yellow spheres arranged in a ring represent the aphid species in a community inhabiting an abandoned field in the south of England. The volumes of the spheres represent the relative densities of the aphid species. Not all aphids are attacked by primary parasitoids but where they are the interaction is represented by green bars connected to brown spheres, the latter representing different primary parasitoids. The width of the bars and the size of the brown spheres represent the relative abundances of primary parasitoids (on a different scale to aphid abundances). Secondary parasitoids (red spheres) have trophic links (blue bars) to primary parasitoids. Again the thickness of bars and size of spheres represent the relative abundance of secondary parasitoids (on their scale).

The structure of aphid food webs has been explored, in particular, by the construction of quantitative food webs in which the density of all species and interactions are given in common units [3,4,20] (figure 2). These observational studies have informed the design of experiments to test the existence and importance of apparent competition and other indirect effects in the field [21,22], extinction cascades in experimental cage populations [23–25] and the effect of possible climate change on food web structure [26].

### (b) How representative are aphids?

We have outlined numerous advantages of using aphids to investigate the impact of symbionts in food webs, but are aphids a symbiont-rich anomaly or good models of insect–microbe associations across the insects? Nutritional primary symbiosis appears to be ubiquitous in those insects that feed exclusively on nutrient-limited diets, such as phloem, xylem and vertebrate blood [6]. In many systems, the microbial partners are bacteria, but endosymbiotic fungi (reviewed in [27]) and gut-dwelling protists (reviewed in [28]; these protists themselves have bacterial symbionts) are also known. Aphids have only a single primary symbiont, but some insects require two or more symbionts to supplement the diet successfully, with some elegant examples of complementary biosynthesis [29–31]. Nevertheless, the aphid–*Buchera* system seems in many respects to exemplify the relationship between an insect and an obligate nutritional symbiont, including genome reduction of the symbiont, localization to a discrete organ and exclusively maternal transmission [32]. We note that not all obligate symbionts are nutritional: both reproductive parasites [33] and defensive symbionts [34] have apparently made the transition to become indispensable to their host. A particularly interesting example is the case of *Wolbachia*, which is essential for normal reproduction in the parasitic wasp *Asobara tabida* [33]. *Wolbachia* frequently distorts host reproductive biology and hosts may evolve measures to counteract the manipulation. The *status quo* may thus be a balance of manipulation and counter-manipulation with removing the symbiont resulting in dysfunction.

Facultative symbionts are much less well investigated than primary symbionts across insect groups, although estimates (which include reproductive parasites such as *Wolbachia* and *Cardinium*) suggest that well over one-third of all insects are infected [1,35]. It is, therefore, difficult to say with certainty whether aphid secondary symbionts are representative of facultative symbionts more broadly. Defensive microbial symbiosis occurs across multiple insect taxa, is provided by a broad range of bacterial groups and acts against a broad range of different natural enemies; it seems likely that researchers have so far discovered only a small minority of protective symbioses [1]. Aphids may well host a greater diversity of protective facultative symbionts than other insect groups, with seven species of defensive symbiont described from pea aphids alone, but the principles drawn from studying aphid symbionts in food webs should still translate to other, less diverse, systems.

## 2. Symbiont effects on interactions: what roles do they play within food webs?

Symbionts impact the biology of their insect hosts in a variety of ways which can affect interactions at lower and higher trophic levels and thus potentially shape food web structure. Here, we consider how the different phenotypic effects of symbionts may influence food web structure and dynamics.

### (a) Abiotic stress

Endosymbionts can influence their host's fundamental niche by modifying their resistance to abiotic stressors. This can allow their hosts to colonize new habitats or extend their geographical range. For example, *Buchnera* can be damaged by high temperatures with negative effects on their hosts and this is suspected to limit the geographical range of aphids which are chiefly temperate insects [36,37]. The secondary symbionts *Serratia symbiotica* and X-type have been shown to help protect aphids against heat shock [38–40] and the frequency of *Serratia* is higher in arid compared with temperate regions [16]. The exact mechanism of protection is not known but it is thought that *Serratia* might produce compounds (such as chaperones and heat-shock proteins) that ameliorate the heat damage to *Buchnera* [41] or it might compensate for the metabolic function usually performed by the damaged *Buchnera* [42].

Not all secondary symbionts help their host withstand thermal damage. Carrying *Regiella insecticola* makes aphids more susceptible to heat shock [39]. *Hamiltonella defensa*, as will be discussed below, confers protection against parasitoids but this fails under heat stress, though to a lesser degree in the presence of X-type [10,43]. Outside aphids, the phenotypic effects of other symbionts can be temperature-dependent; for example, the *Wolbachia* male-killing phenotype in *Drosophila bifasciata* is weaker at higher temperature, an effect that seems associated with reduced bacterial density [44].

The acquisition of new symbionts may thus allow aphids to move into new climatic zones, or limit their ability to do so, and so determine their presence or absence in particular food webs. Symbionts also introduce abiotic context-dependency into host interactions with other organisms (discussed below) and so could contribute to changes in food web structure along environmental gradients.

### (b) Interactions with food plants

Symbionts can affect their herbivorous hosts' ability to use particular plant species or plant parts. The role of obligate symbionts in permitting insects to feed on food resources that are nutritionally deficient, such as plant sap and vertebrate blood, has been understood for over 50 years [45]. Although the capacity to feed on plant sap is enabled by symbionts, a primary symbiont that has become specialized to supplement components of the diet missing in the phloem of a particular plant species may also constrain an insect's potential range of food plants [46]. There is evidence that there are costs to carrying some aphid secondary symbionts that are manifest on some but not on all potential food plants, something that may influence host plant usage in the field [47–49].

The taxon referred to as the pea aphid is actually composed of a series of host-specialized races or biotypes that are to different extents genetically differentiated and specialized on different legume (Fabaceae) host plants [50,51]. There are a number of strong associations between particular biotypes and different secondary symbionts. For example, the symbiont *R. insecticola* is very common on clover (*Trifolium pratense*) in populations throughout the world [52–54]. Tsuchida *et al.* [55] found that removing *Regiella* reduced the capacity of a clone of pea aphid to feed on clover, while the introduction of the same symbiont isolate into a naive aphid host (*Megoura crassicauda*) improved its performance on the same plant [56]. However, other studies have failed to find these effects, which seem, therefore, to depend on the specific genotypes of the aphid and/or bacteria involved [49,57,58]. More generally, evidence from the phylogenetic analysis of pea aphids and their secondary symbionts suggests that the acquisition of symbionts often accompanies host shifts [16], but this work cannot distinguish whether this is linked to host utilization or to other ecological factors correlated with transition to a new host (see also below). The exact role of secondary symbionts in influencing pea aphid host plant use needs further research but elsewhere there is some very convincing evidence of symbionts determining host plant range. In another aphid species, *Aphis craccivora*, the symbiont *Arsenophonus* has been shown to confer plant-specific benefits to its host [59]. Outside aphids, in *Megacopta* stinkbugs, experiments involving the mutual exchange of obligate gut symbionts between species have shown that symbionts are the primary determinants of host plant range [60].

**Figure 3.**
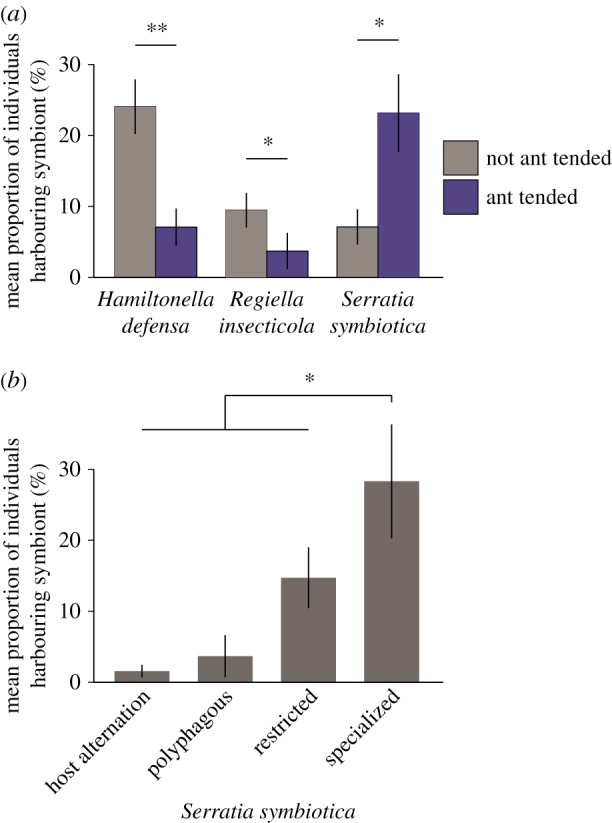
(*a*) Frequency of three pea aphid secondary symbionts in relation to presence of ant-tending. (*b*) Frequency of *Serratia symbiotica* in relation to the host plant range of aphids (from Henry *et al*. [16]). (Online version in colour.)

Comparative phylogenetic studies of a broad range of aphid and symbiont species have thrown up some intriguing patterns that may result from symbiont effects on host plant use. Several phylogenetically distant aphids that feed on the same host plant share similar strains of *S. symbiotica* [15]. Possibly aphids in the same ecological niche are more likely to be infected by the same symbiont but experiments to see whether the symbiont affects performance on this host plant would be interesting. Another intriguing pattern is that *Serratia* is disproportionately uncommon on host-alternating or polyphagous aphid species, possibly indicating a species that needs to adapt to a stable metabolic milieu [15] (figure 3*b*).

The host plant range of herbivores has consequences for food webs beyond simply broadening or narrowing the number of producer species consumed. For example, increasing the diversity of food plants has been shown to decrease the proportion of aphids consumed by predators in food web microcosm experiments [61]. Increasing host range may also (albeit temporarily) move an insect into enemy-free space [62]. Symbionts that influence host plant use, therefore, have the potential to affect both the strength and number of interactions within a food web.

### (c) Interactions with natural enemies

#### (i) Pathogens and parasites

Several species of fungal pathogens are among the most important natural enemies of pea aphids [4] and have been used as biocontrol agents against pest species [63,64]. Prior to the discovery of the importance of secondary symbionts, substantial between-clone genetic variation in fungal resistance had been reported [65], but much of this was subsequently found to be explained by the presence and absence of secondary symbionts. *Regiella insecticola* provides substantial protection against the fungal pathogen *Pandora neoaphidis* [66], and this protection was later found to extend to another species of aphid specialist fungi but not to a generalist fungal pathogen [67]. Several other species of unrelated bacterial symbionts have also been found to provide protection against *Pandora* in the pea aphid [68] and *Regiella* has been shown to be protective in other aphid species [69]. The mechanistic basis of this resistance, and whether different symbionts use the same mechanism (either through convergence or horizontal transfer), is not yet known. In other systems, symbionts are also known to provide protection against pathogens and parasites. For example, the bacterial symbiont *Wolbachia* protects *Drosophila melanogaster* against RNA viruses [70] while *Spiroplasma* can protect *D. melanogaster* from parasitic nematode infection [71].

#### (ii) Parasitoids and hyperparasitoids

Multiple species of aphid endosymbiont play a role in protecting their host against parasitoids. Aphid parasitoids are all solitary koinobiont (allowing their host to continue to develop and increase in size after parasitism) endoparasitoids, ovipositing and completing larval development within a living aphid. This development may be prevented at the egg or larval stage by the presence of the symbiont *H. defensa* [72], although some aphid clones also show different degrees of intrinsic resistance to parasitoids in the absence of protective symbionts [73]. Even if a wasp successfully emerges from an aphid carrying *H. defensa,* it often is of reduced size and fitness [74]. Other symbionts have also been shown to improve aphid resistance to parasitoids: *S. symbiotica* is mildly protective [75], and the bacterium known as X-type enhances protection in co-infections with *H. defensa* [10,40]. In *Myzus persicae*, a strain of *R. insecticola* has been shown to protect against parasitoids, and this phenotype persists when bacteria are transferred to other aphid species via artificial symbiont injection [76].

Symbiont-mediated protection in aphids is effective against a range of different hymenopteran parasitoids, although strains differ widely in their efficacy against different wasp species [77–79]. Work on the parthenogenetic wasp *Lysiphlebus fabarum* attacking black bean aphid (*Aphis fabae*) has shown that the extent of protection can depend quite finely on the precise genotypes of the wasp and symbiont involved [80,81]. The protection provided by symbionts also depends on the age at which the aphid is attacked [74], probably linked to increasing symbiont titre with aphid maturity.

Aphid primary parasitoids are attacked by a number of so-called ‘secondary’ parasitoids, including both ‘true’ hyperparasitoids which attack the larval parasitoid, and ‘mummy’ parasitoids which attack only after the aphid has died and the primary parasitoid pupated (the remaining dried husk of the aphid is termed a ‘mummy’) (reviewed by Sullivan & Völkl [19]). There are as yet no data available on how the presence of protective symbionts might impact hyperparasitoids at the fourth trophic level. However, secondary parasitoids are very likely to be affected by the presence of symbiont-mediated resistance in aphid population as this would reduce the absolute availability of primary parasitoid hosts. It would also potentially change the species composition of the primary parasitoids available, to a particular hyperparasitoid species' advantage or disadvantage depending on its host range. Given that some protective symbionts are found more frequently on aphids feeding on certain host plants or in certain habitats, the spatial distribution of potential hosts will also be affected. Finally, where aphid symbionts act on the primary parasitoid later in development, there is also a potential additional cost for true hyperparasitoids: the time spent investigating potential hosts and the number of eggs laid in ultimately unsuitable hosts.

Symbiont-mediated resistance against parasitoids will affect host–parasitoid food web structure by disallowing some trophic links entirely, or, and probably more likely, by reducing the strength of others. The intraspecific differences in susceptibility to parasitoids that result mean that observed rates of successful parasitism in the field may underestimate parasitoid attack rates. The advantages of carrying protective symbionts will vary with parasitoid attack rate and if in the absence of parasitoids there are costs to symbiont infection then there is the potential for dynamic cycles in parasitoid abundance and symbiont frequency [82] which might explain why protective symbionts are facultative rather than obligate. There is also the potential for the specificity of symbiont defence [77–79] to influence competitive interactions between parasitoid species, for example, by improving the success of species that are otherwise inferior competitors. Although best-studied in aphids, symbiont-mediated protection against parasitoids is also found in other systems, for example, a *Spiroplasma* species in *Drosophila hydei* has been shown to increase survival following attack by the endoparasitoid *Leptopilina heterotoma* [83,84], and it seems likely that other examples will be discovered. Finally, parasitoids themselves can also carry bacterial symbionts that alter prey selection behaviour [85].

#### (iii) Predators

Aphid populations are subject to intensive attack by predators and predation is the fate of most hosts carrying symbiont. Were symbionts able to reduce predation rates, it would be both highly advantageous to their hosts and important for food web structure. In a recent study, predators that fed on aphids with symbionts suffered increased mortality relative to those feeding on aphids without symbionts, although no effects on the rates of predation of aphids with and without symbionts were observed [86]. There is some limited evidence that the presence of the symbiont *Rickettsiella* might be correlated with a decreased risk of predation [87]. However, aphids infected with *H. defensa* display reduced anti-predator behaviour such as kicking and dropping, which is predicted to increase mortality from predators [88]. Outside aphids, there is at least one very clear example of a symbiont providing protection against predation: a *Pseudomonas* bacterium synthesizes toxins that protect its host, the rove beetle *Paederus sabaeus*, from predators [89,90]. The *Proftella* symbiont of the psyllid *Diaphorina citri* is likewise thought to synthesize defensive toxins [34]. Overall, symbionts seem less likely to provide protection against predators compared with parasites or parasitoids which have a more intimate association with their host.

### (d) Competitors

There are two main ways in which symbionts might influence non-trophic interactions between herbivores. First, symbionts may be costly to their host and reduce their competitive ability. Experimental laboratory studies suggest that aphids with secondary symbionts may be outcompeted by conspecifics not carrying symbionts in the absence of the selection pressure (for example, parasitoid presence) that favours the particular symbiont [91,92]. There is no reason to think that this would not also apply to interactions between species. It would be interesting to model the outcome of competition between two species (or in aphids genotypes) carrying facultative endosymbionts to explore their joint frequency and density dynamics, especially in circumstances when symbiont carriage is costly or provides other benefits.

The second way in which symbionts could affect interactions between herbivores is via symbiont-mediated protection leading to apparent mutualism or competition. Apparent mutualism occurs when the presence of a resistant but attractive alternative host (or prey) leads to reduced natural enemy success and hence lower densities to the benefit of both host species [93]. An aphid with a protective symbiont might thus provide an indirect benefit to another species with which it shares a parasitoid or parasite. Were this second species to be a resource competitor then this would undermine the value of the protection conferred by the symbiont. There is some evidence that parasitoids can tell whether a potential host carries a protective symbiont [94]. If this causes a parasitoid to switch to its alternative host then the apparent mutualism becomes apparent competition. If the alternative host is also a resource competitor then the symbiont's host gets a second indirect benefit from carrying the protective symbiont.

## 3. Patterns of symbiont distribution within and across species

Large-scale surveys of secondary symbionts in aphids [15,16] have revealed a variety of patterns suggesting that symbiont loss and gain may be associated with different ecological factors. As yet it is difficult to untangle the causal pathways underlying these associations but they do emphasize the ecological community context in which aphid–symbiont associations emerge.

As discussed above, the pea aphid is composed of genetically differentiated host biotypes that are adapted to different food plants [51] across which symbionts are distributed non-randomly. For example, the symbiont *H. defensa* is found at particularly high frequencies in the aphid populations that feed on the plants *Medicago sativa*, *Ononis spinosa* and *Lotus pedunculatus*, while pea aphids that feed on *Lathyrus* species are rarely infected with any facultative symbionts [53]. The strong association between *R. insecticola* and clover (*Trifolium* spp.) has already been mentioned.

There are geographical patterns in symbiont distribution. For example, *H. defensa* is absent in pea aphid populations from Asia, Australia and South America, but is common in populations from Europe and North America [95]. Pea aphid is an Old-World species moved round the globe by humans. Do these patterns reflect the contingencies of introduction and founder effects or different ecological pressures in the different regions? *Serratia symbiotica* is a common symbiont of aphids feeding on cultivated pea *Pisum sativum*; in the Middle East it can reach a prevalence of 70% compared with as low as 27% in parts of Europe [16]. The ability of *Serratia* to help its host withstand heat shock (see above) may be responsible for this pattern. *Regiella insecticola* was found to be more common in northern regions of Japan, which have cooler climates with greater annual precipitation [52], perhaps more conducive to the fungal infections against which *Regiella* provides protection. Variation in the presence of facultative symbionts across geographical regions has also been documented in the cowpea aphid, *A. craccivora* [96], and in other aphid species (reviewed in Zytynska & Weisser [95]), as well as in whiteflies [97].

The distribution of symbionts across aphid populations is determined by the joint action of horizontal and vertical transmission. Symbionts cross species boundaries by horizontal transmission and can similarly move among populations and lineages within a species. We do not understand how horizontal transmission occurs in nature but transmission mediated by parasitoids has been demonstrated in the laboratory [98] and whitefly symbionts very closely related to those occurring in aphids can be transmitted through the host plant [99]. Once in a new host, the presence of a symbiont can provide an advantage to the aphid matriline, allowing it to increase in frequency in the aphid population. Symbiont spread will also be subject to drift (neutral increases and decreases in frequency) as well as loss during vertical transmission. Most estimates of the frequency of vertical transmission are near one in the asexual generation but there is some evidence that it may be lower in the more poorly studied sexual overwintering generations [100].

Henry *et al.* [16] surveyed the symbionts in over 1000 collections of pea aphid from around the world and built twin phylogenies of both host and bacteria. The joint effects of horizontal and vertical transmission can be seen as structuring this dataset. For example, mapping *R. insecticola* presence onto pea aphid matriline phylogenies shows that this symbiont was acquired on a relatively small number of occasions and transmitted to many of its descendent lineages, though its absence from some is evidence of symbiont loss. *Hamiltonella* shows a similar pattern but with a greater number of introductions while, by contrast, there are few lineages where *Serratia* is found at high prevalence. On top of these patterns, sporadic occurrences of symbionts often occur at the tips of host phylogenies, possibly reflecting a flux of short-lived symbiont infections. The same symbiont isolates have been repeatedly acquired by certain pea aphid biotypes through horizontal transmission prior to colonizing certain ecological niches, such as new plants and geographical regions [16]. Although such analyses cannot demonstrate causality they do identify patterns consistent with existing hypotheses (such as the role of *Serratia* in protecting their hosts from temperature extremes in hot climates) as well as generate new hypotheses for experimental investigation (such as associations between particular symbiont–host plant pairs).

We still know little about why certain host species are more likely than others to harbour facultative symbionts but comparative work suggests that the presence of symbionts can be strongly influenced by the life-history traits of their hosts [15]. For example, aphid species that are protected by ant mutualisms are less likely to harbour symbionts that provide protection against natural enemies (figure 3*a*), possibly because ants reduce pressures from natural enemies, and protective symbionts are, therefore, not required [15]. If this explanation were correct, it would be an example of facultative symbiont distributions being shaped by the community interactions of the host.

## 4. Food webs within food webs

**Figure 4.**
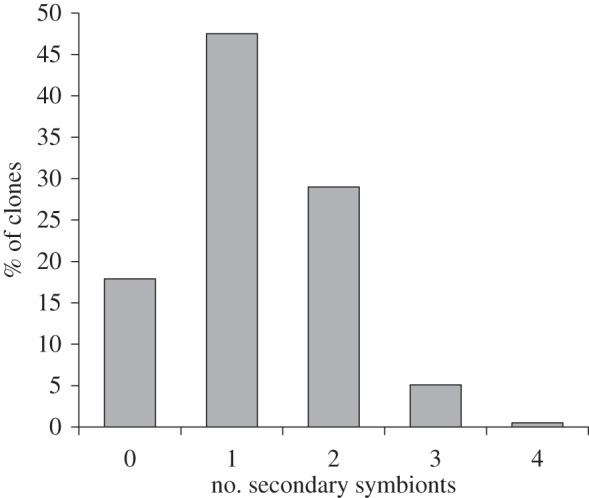
The percentage of 1104 pea aphid collections from around the world that harboured 0–4 species of secondary (facultative) symbionts (adapted from Henry *et al*. [16]).

The majority of work on insect symbionts has, by necessity, studied host–microbe pairings in isolation. However, studies of symbiont distribution and abundance in natural populations have provided many examples of co-infections between multiple species and strains of symbionts [101,102] (figure 4). This is perhaps surprising, because theory predicts that within-host competition among symbionts (especially if this also entails cost to hosts) will tend to lead to just one symbiont persisting [103], and because transmission of endosymbionts is imperfect. Furthermore, the patterns of co-infection uncovered in these studies indicate that some combinations of endosymbionts occur less or more often than would be expected by chance [16,53,101,104], suggesting that selection shapes patterns of co-infection. If within-host interactions among symbionts impact the ecologically relevant phenotypes of symbiont infection, then their dynamics will affect community interactions of the host.

Hosts offer limited resources to symbionts and symbionts successful at competing for resources should increase in frequency within hosts. Evidence for such competitive interactions among co-infecting symbionts comes from longitudinal studies of fluctuations in within-host symbiont titres. For example, in aphids, infection with *Serratia* suppresses titres of the primary symbiont *Buchnera* [42]. Similarly, *Wolbachia* densities in *D. melanogaster* were found to be lower in flies co-infected with *Spiroplasma*, suggesting that *Spiroplasma* is negatively influencing *Wolbachia* growth [105]. These competitive interactions are thought to be costly for hosts. Aphids that are co-infected with *Serratia* and *Hamiltonella* have high *Serratia* densities relative to single infections, which may explain why aphids harbouring a double infection have reduced fecundity [106], although other studies [68] have found no fitness costs of co-infection.

Despite the potential for competitive exclusion, symbiont co-infections are common. In pea aphids, for example, individuals have been found carrying four facultative symbionts in addition to the primary symbiont *Buchnera* [54]. There are several potential processes that may help explain the existence of these multiple associations. First, symbionts may have different positive and complementary effects on their hosts which result in higher fitness of hosts with multiple infections than either single infection can produce alone. Second, symbionts can be localized in different host tissues so that costs to the hosts are reduced. Tissue differentiation has been suggested as an explanation for the coexistence of multiple strains of *Rickettsia* found in individual whiteflies [107]. Third, symbionts that persist through reproductive manipulation can coexist within a host lineage by using different manipulation strategies. Fourth, symbionts might have synergistic effects on their host's phenotype. For example, joint infections of *Hamiltonella* and *Serratia* are more costly to their hosts than single infections but appear to provide stronger protection against parasitoid wasps [106].

How might the within-host dynamics of endosymbionts affect the population dynamics of their host and the species with which it directly and indirectly interacts? First, co-infections may produce novel phenotypes—for example, allowing a protective phenotype to persist in otherwise unfavourable abiotic conditions [10]—which can then impact on food web interactions. Second, acquiring an additional symbiont species may rescue an existing phenotype. Where genetic exchange between bacterial symbionts is rare, there is a risk that ecologically-relevant phenotypes of symbionts will be lost due to the continuous accumulation of deleterious mutations (Müller's ratchet); a complementary symbiont may replace this lost function. For example, *Buchnera* in the aphid *Cinara cedri* lack certain amino acid synthesis pathways that are present in *Buchnera* of other aphid species. However, *C. cedri* obligately harbours *S. symbiotica* that appear capable of supplying those specific amino acids [108–110]. The precise sequence of function loss in *Buchnera* and substitution by *Serratia* is not yet clear, but maintenance of the phenotype (feeding on phloem) requires both symbiont species. Complex examples of nutritional complementarity by multiple symbionts have now been described in other sap-feeding insects [29,30,111] but whether such complementarity might exist for defensive phenotypes is as yet unknown. Finally, and more speculatively, the presence of one symbiont might prevent the acquisition of another, if the second is inferior in within-host competition. In that case, the ability to adapt to changing ecological conditions by acquiring new symbionts from the ‘horizontal gene pool’ might be constrained.

## 5. Lessons from *Wolbachia*

*Wolbachia* is the most common and widely distributed endosymbiotic bacterium and provides an interesting contrast with the endosymbionts we have been discussing in aphids. *Wolbachia* is best known for its ability to manipulate host reproduction [112] and it infects 40–60% of all insect species at varying frequencies [113,114]. *Wolbachia* are transferred horizontally at relatively high rates, so that there is little phylogenetic or geographical structure in *Wolbachia*–arthropod associations [115].

*Wolbachia* can manipulate host reproduction in a number of ways [116] which all result in symbiont-infected females producing more daughters than their uninfected counterparts. Three strategies—feminization, parthenogenesis induction and male-killing—all do this directly by manipulating the sex ratio with population level consequences [117]. Cytoplasmic incompatibility (CI), by contrast, disadvantages uninfected females by preventing them from successfully mating with males that carry *Wolbachia* [112]. This disadvantage is frequency dependent and when *Wolbachia* carriage carries cost there is a threshold infection frequency that must be exceeded before spread occurs. Because CI caused by *Wolbachia* is strain-specific, the acquisition of multiple strains can lead to population splitting and speciation. *Wolbachia* spread through CI can cause a mitochondrial sweep as the mitochondrial variant associated with the initial infection hitch hikes to high frequency or fixation [112,116]. *Wolbachia* can thus compromise the use of mitochondrial genetic markers for species delimitation, including the commonly used DNA barcode fragment of the COI gene. For example, if *Wolbachia* is initially introduced into a new species through a rare interspecific mating event then the mitochondrial type of the second species can replace that of the first. There are a number of examples of where this has undermined standard DNA barcoding [118], though it does not appear to occur commonly enough to compromise its widespread use [115,119]. It would be beneficial to include endosymbiont screening in DNA barcoding studies to help assess the importance of CI endosymbionts in speciation. *Wolbachia* is not the only bacterium that spreads through reproductive manipulation—other common endosymbionts including *Cardinium*, *Rickettsia*, *Spiroplasma* and *Arsenophonus* use similar strategies [116].

Although long known for reproductive manipulation, recent research on *Wolbachia* has suggested that they also can also give rise to host phenotypes that resemble those produced by the aphid secondary endosymbionts described above. For example, *Wolbachia* can protect insects against RNA viruses [70,120], although enhancement of viral infection has also been recorded [121]. Where this phenotype occurs in the insect vectors of mammalian and human diseases, it can have far-reaching effects on community ecology and human health: *Wolbachia* has been introduced in the laboratory into the main vector of dengue virus where it suppresses transmission; insects from these cultures have then been released in the field so successfully introducing the beneficial symbiont into wild populations [122]. *Wolbachia* has also been shown to protect mosquitoes against nematodes [123] and to provide mild protection for *D. hydei* against parasitoids [83,84], although a case of reduced resistance has also been recorded [124].

*Wolbachia* are found in parasitic nematodes as well as insects. In nematodes they are obligate mutualists with phylogenies exactly concordant with their hosts [112], like the aphid primary endosymbiont *Buchnera* described above. Like most obligate symbionts they have a nutritional function and also play a role in helping the nematode evade the immune response of their vertebrate hosts [125]**.** A rare example of *Wolbachia* having a nutritional role in an insect host is the obligate symbiosis of *Wolbachia* and bed bugs (*Cimex*), where it provides its host with vitamin B [126,127]. *Wolbachia* has the widest range of phenotypes recorded for any symbiont. Possibly it is unusually versatile, but alternatively it may just be comparatively well studied. If the latter is true, which is our suspicion, it suggests that more intensive study of other symbionts will reveal important new biological phenotypes. This makes it even more important to consider the possible effects of this group of organisms in food web studies.

## 6. Barcoding and symbiont biology

The DNA barcoding revolution has important implications for symbiont biology, especially for studies attempting to move beyond particular host–symbiont interactions to understanding how symbionts are distributed across, and move through, their hosts' communities.

The most obvious and already realized benefit is the ease with which communities can be described taxonomically. This can save considerable time even in regions that are taxonomically comparatively well known such as northern Europe [128]. However, in other more poorly known regions it can be transformational and allow community ecology to be undertaken that would be logistically impossible otherwise without unrealistic investment in primary taxonomy.

Eukaryote symbionts such as yeast and other fungi can also be identified using cytochrome *c* oxidase 1 (COI) barcodes though reference sequences for these groups are still comparatively scarce; the nuclear ribosome internal transcribed spacer (ITS) is likely to prove a more useful candidate for fungal barcoding [129]. The bacterial ribosomal 16S gene is a *de facto* barcode for prokaryote symbionts, while multilocus sequence typing schemes have been developed for symbionts such as *Wolbachia* [130] and the aphid symbionts discussed here [15] to explore within-genus genetic structure.

At the moment the use of host and symbiont barcodes is still limited by costs (though these have reduced dramatically in recent years) and by the human labour needed for extraction and sequencing. Robotic solutions to the high-throughput analysis of very large number of specimens are possible now using current technology, though the rather niche application to community ecology has not justified the expense required for their development. General advances in robotics and their application to other areas of biology are likely to remedy this and provide the means for community ecology to enter the era of ‘big data’—an exciting prospect.

The barcoding movement has now obtained DNA sequences from approximately 2.5 million specimens of approximately 200 000 species [131] and for a large fraction of these specimens DNA has been archived in storage. Many of these specimens are insects and this is a potentially very valuable resource for exploring symbiont distribution across large assemblages of species. One drawback, however, is that often the DNA is only extracted from the insect leg. Insect symbionts tend to be found in the abdomen, often associated with specialist structures near the reproductive organs, and though they do occur in the haemolymph their densities in peripheral organs are likely to be low.

Large-scale barcoding projects such as the Área de Conservación Guanacaste survey in Costa Rica and the Global Malaise project [131,132] are sampling intensively in restricted areas whole ecological communities. Related projects such as the Island Digital Ecosystem Avatars (IDEA; http://mooreaidea.org/) project on Moorea in the Pacific are doing similar things with a metagenomic perspective. With the proviso that appropriate DNA material needs to be stored, such projects offer the exciting prospect of surveying symbiont distribution across entire communities, while metagenomic approaches will make it easier to discover novel symbionts that would be missed by targeted surveys, as well as understand better the dynamics of genetic transfer among symbionts and hosts. Even where this may not be presently possible, we believe there is strong argument for extracting and archiving DNA resources in anticipation of a time when the rate limiting step in this area of science is likely to be the collection of material rather than the molecular biology and bioinformatics.

## 7. Conclusion

We believe that the two fields of insect symbiosis and food web ecology are ripe for fruitful contact to their mutual benefit: understanding the establishment and maintenance of symbioses needs to be pursued within a food web context, and the evidence that symbionts can play a pivotal role in the interactions that shape food webs is now strong.

The ecological and evolutionary interests of symbionts and their hosts are inseparable although not always perfectly aligned. This means that symbionts cannot be viewed as independent agents—just further species—within a food web, but neither can they be treated as another species trait or source of intraspecific variability. Instead they occupy an intermediate position, subject to the same intense selection pressures as nuclear traits that affect and are affected by their host's biotic and abiotic environment, but which can often move horizontally between species in the same or possibly different trophic levels. There is of course considerable biological variation within symbionts, from those that are obligate and only transmitted vertically and whose population biology is identical to maternally transmitted organelles, to symbionts that harm their hosts and are transmitted horizontally at relatively high frequency and which more resemble pathogens or parasites. Nevertheless, many of these various symbionts play roles that both are impacted by food web interactions, and will in turn impact upon them, one obvious example being symbiont-mediated defence and natural enemy pressure.

Experiments in microcosms and mesocosms have proved valuable in exploring the processes underlying the persistence of insect food webs. For example, Sanders & van Veen [25] and Sanders *et al*. [24] have shown in model aphid–parasitoid communities how the removal of one key species can lead to extinction cascades affecting many others. Working with this group [133], we have recently explored whether introducing a defensive symbiont and so disrupting a specific host–parasitoid interaction can have similar cascading effects. We showed that releasing a competitive dominant from parasitism could have cascading effects on inferior competitors and their natural enemies. In general, we suggest that experimental community ecology can be used to test hypotheses about how symbionts may structure food webs. As the more straightforward impacts of symbionts in food webs become better understood, it will be possible to investigate whether defensive symbionts, in particular, can influence more complex food web properties such as stability and linkage richness.

Modern molecular tools provide the opportunity for the first time to characterize the symbionts present in real communities in natural environments. In well-characterized systems reliable high-throughput diagnostic polymerase chain reaction (PCR) can be used to screen large numbers of individuals for multiple symbiont species. Systems that are less well characterized can be investigated efficiently using next generation sequencing methods. However, at the moment it is not possible to predict the joint phenotype of insect plus symbiont from sequence data alone, though this may become feasible as we understand more about the mechanisms involved. This will help explain why seemingly minor genetic variation in the host or symbiont, and their interaction, can have a major effect on the phenotypes that influence food web dynamics.

Laboratory studies of the costs and benefits of carrying symbionts sometimes produce results that are at odds with their observed distribution in the field. Symbiosis researchers have acknowledged a need for more field studies of costs and benefits [134], especially those that consider interactions with the broader community or organisms that interact with a focal species [95]. For example, competition between fungal pathogens and parasitoids of aphids is known to be asymmetric, with fungal pathogens killing the developing parasitoid along with its aphid host [135]. The presence of a secondary symbiont that protects against fungal pathogens [66,136] would, therefore, also protect the developing parasitoid. Would parasitoid mortality outweigh any advantage from pathogen protection, and would improved parasitoid survival have population consequences for the symbiont-bearing aphid as well as other clones or species with which it might compete?

To conclude, facultative symbionts such as those that can be transmitted between aphid species have been thought of as a horizontal gene pool from which species can sample potentially useful adaptations. These symbionts link together the evolutionary futures of the species they move among. We argue that symbionts, by affecting food web interactions and structure, can also influence the interactions between species, so influencing their ecological futures. The evolutionary play in the ecological theatre [137] thus gets another twist.
